# Targeting targeted agents: open issues for clinical trial design

**DOI:** 10.1186/1756-9966-28-66

**Published:** 2009-05-22

**Authors:** Emilio Bria, Massimo Di Maio, Paolo Carlini, Federica Cuppone, Diana Giannarelli, Francesco Cognetti, Michele Milella

**Affiliations:** 1 Department of Medical Oncology, 'Regina Elena' National Cancer Institute, Rome, Italy; 2 'G. Pascale' National Cancer Institute, Naples, Italy

## Abstract

Molecularly targeted agents for the treatment of solid tumors had entered the market in the last 5 years, with a great impact upon both the scientific community and the society. Many randomized phase III trials conducted in recent years with new targeted agents, despite previous data coming from preclinical research and from phase II trials were often promising, have produced disappointingly negative results. Some other trials have actually met their primary endpoint, demonstrating a statistically significant result favouring the experimental treatment. Unfortunately, with a few relevant exceptions, this advantage is often small, if not negligible, in absolute terms. The difference between statistical significance and clinical relevance should always be considered when translating clinical trials' results in the practice. The reason why this 'revolution' did not significantly impact on cancer treatment to displace chemotherapy from the patient' bedside is in part due to complicated, and in many cases, unknown, mechanisms of action of such drugs; indeed, the traditional way the clinical investigators were used to test the efficacy of 'older' chemotherapeutics, has become 'out of date' from the methodological perspective. As these drugs should be theoretically tailored upon featured bio-markers expressed by the patients, the clinical trial design should follow new rules based upon stronger hypotheses than those developed so far. Indeed, the early phases of basic and clinical drug development are crucial in the correct process which is able to correctly identify the target (when present). Targeted trial designs can result in easier studies, with less, better selected, and supported by stronger proofs of response evidences, patients, in order to not waste time and resources.

## Introduction

The increasing amount of knowledge about biological targets is nowadays going to switch the balancing and equilibrium between the medicine for the 'entire population' and the medicine for 'the individual', in favour of the latter, in order to better aim to a modern concept of 'ideal medicine'. The results obtained with the traditional clinical trial design with molecularly targeted agents so far are far from being optimal. Indeed, with the exception of trastuzumab for breast cancer, we observe 4 common outcome patterns of randomized trials in solid tumors: 1) studies reporting a significant while small survival benefit for the targeted agent (advanced pretreated non-small-cell lung cancer, NSCLC, erlotinib versus placebo) [1]; 2) studies reporting a significant while minimal survival benefit for the targeted agent (advanced untreated pancreatic adenocarcinoma, erlotinib plus gemcitabine versus gemcitabine) [2]; 3) studies reporting no significant differences in survival (advanced pretreated NSCLC, gefitinib versus placebo) [3]; and 4) studies reporting an unexpected significantly detrimental effect of the targeted agent (locally advanced NSCLC, maintenance gefinitib after chemotherapy versus placebo) [4]. Given these scenarios, no major differences in the trials results with (old) and so-considered 'un-targeted' chemotherapeutics do appear, with the exception of trastuzumab.

## Targeted versus untargeted design for new drugs

What is wrong with this design approach when molecularly targeted agents are tested? The 'new age' of medical oncology is experiencing many biological advances and discoveries from the basic science side and the new available techniques, concurrently with the release of new available drugs. Moreover, medical oncology represents the field of clinical medicine with the higher failure-rate for late-stage clinical trials, when compared to the other specialties, and with the higher time- and resource-intensive process, with more than 800 million US dollars to bring a new drug to market.

So, the clinical trial design methodology needs to be updated, given the 'confusion' provided by the discovery of new targets, which identify (in many cases) new patient' subgroups. For these reasons, it seems reasonable to ask ourselves, among a high number of other relevant questions, if: 1) response rate is an adequate end-point for phase II trials with molecularly targeted agents; 2) the randomized phase II fashion represents a real step beyond; and 3) which kind of phase III do we need. On the other hand, we should restrict this 'revision' of the design approach to those drugs with a known targeted population (and so apply a 'targeted-design'), and do not discard the traditional way for drugs without a clear beneficial patient' group (and so apply an 'untargeted-design').

The metastatic breast cancer scenario do offer both options: the trastuzumab and the bevacizumab registration trials [5,6]. Trastuzumab entered the market thanks to a relatively small trial (469 patients), while able to determine a huge survival difference (5 months); if a traditional untargeted design would have been adopted, considering a 20–30% prevalence of the HER-2 positive population, and a treatment effect of 10% benefit, more than 23 thousands of patients would have been required [7]! Conversely, although the untargeted approach used for bevacizumab allowed to register the drug with a significant (while absolutely small) benefit in progression-free survival, retrospective evidences are emerging indicating those subset of patients where the benefit is maximized, on the basis of genetic variants [8].

## The role of 'early phases': are traditional phase I studies with new drugs reliable?

Traditional phase I studies for chemotherapeutic agents are designed to find the maximum tolerated dose (MTD) and the dose-limiting toxicity (DLT) of the drugs. The assumptions underlying phase I designs are that for most cytotoxic agents there is a direct relationship between the dose of a drug, its antitumor effect and toxicity. Therefore, toxicity and activity increase with the increasing of the dose of the drug and there is a recommended dose that provides clinical activity with acceptable toxicity. Thus, toxicity has been seen as a surrogate for potentially effective doses. With biological agents, acting on highly specific targets expressed in cancer cells, the MTD may not be reached if the drug has a much wider therapeutic ratio: therefore, an increase of the doses to toxic levels may be not necessary to achieve the maximum activity and it may be an irrelevant end point. There are alternative end points for these agents that can be usefully employed in phase I studies: the identification of a molecular drug effect (the 'target effect'), the measurement of 'surrogates' for biological activity and the assessment of drug plasma levels. The identification of the 'target effect' through pharmacodynamic assays is proof of principle and can be proof of activity of the drug. The main application of pharmacodynamic studies is to help in the selection of the minimum target inhibiting dose (MTID) and the optimal schedule of administration of a drug [9].

## The role of 'early phases': what about phase II studies?

An 'average' drug development time-process performed by the best multicenter, cooperative, skilled, international group, which is constituted by a 1-year phase I to find the safe dose of the new drug and its toxicities, a 1–2 years formal phase II to test the activity and the tolerability (on the basis of an hypothesis formulated on historical data), and a 2–5 years classical phase III to see how the drug compare with the standard, will result into, at least, a 5 years long course. With this 'favourable' described perspective, it easy to understand that the role of the early phases (preclinical, phase I and II) is crucial in order to have a positive results in the forthcoming phase III. After a good (and independent, unbiased) preclinical development, within the first 1–3 year of the clinical development it is easy to control the drug effect, to monitor either the biological and the clinical action, and to identify the exact target (when present). Moreover, this is the moment when it is possible to screen for all putative surrogate biological end-points. When a drug enter the phase II study, is difficult to obtain all these informations, given the present statistical borders; only stopping rules into pre-planned interim analyses are allowed (with all their related concerns).

What are the limitations in the phase II study design? A single-arm formal phase II is designed upon response limits weighted on the basis of historical data or clinical experience of standard treatment, which constitute the benchmark response rate. The choice of such border is influenced by several biases, according to the recent report by Vickers et al [10]. When appropriate criteria for citation of prior data are fixed, those studies that met them were significantly less likely to reject the null hypotheses (33%) than those cited that did not meet the criteria (33% versus 85%, respectively; p = 0.006) [10]. With this perspective, it seems that the decision to go into a phase III is biased by not accurate reporting of historical data. By this, if wrong hypothesis is tested, the chance of a positive, reliable result into the following phase III is reduced; unbiased evidences with accurate testing hypotheses are needed to improve the success rate of a new drug in a randomized trial [11].

Do we have predictors of success in the subsequent phase III, into the phase II studies? A recent analysis of a series of phase II with molecularly targeted agents reports that the presence of positive results (p = 0.027), the sponsorship of a pharmaceutical company (p = 0.014), the short interval between the publication of phase II and III (p < 0.001) and multi-institutional trials (p = 0.016), are all independent predictors of success at the multivariate analysis [12]. Another important finding (which is commonly reproduced in many phase II studies with molecularly targeted agents) is that if the rate of disease progression is chosen as measure of drug effect instead of the 'classical' response rate, the chance of a positive following phase III is higher [12].

## 'Myths' of targeted agents: activity or efficacy as phase II primary end-point?

At least two 'myths' are perceived to be specific features of molecularly targeted agents. The first one is that, conversely to classical cytotoxics, molecularly targeted agents would selectively hit a specific molecule or enzyme and that their functional and clinical effects would be directly related to the level of target inhibition. A recent exhaustive review by Karaman et al visually shows that the many commonly used TKIs (tyrosine-kinase inhibitors) may hit several intracellular pathways (for example sunitinib), while others really seem to restrict their action upon one proliferation pattern (for example lapatinib), by elegantly using kinase dendrograms [13]. It would be interesting to understand how much the classical cytotoxic differs in such kind of analysis from the so-called 'targeted' agents. Recent reports strongly enhance the potential 'targeting' of old chemotherapeutics [14].

The second 'myth' to discard is that molecularly targeted agents are 'cytostatic' in nature, i.e. they will slow down growth, but seldom shrink pre-existing tumor masses. That seems true for sorafenib in hepatocellular carcinoma, where no major difference in both responses and disease stabilization are present between patients receiving such drug and those undergone placebo [15]. Nevertheless, this trial returns in suggesting that these drugs show much more benefit in efficacy end-points rather than old-classical activity (at least measured as we are used to so far); indeed, the benefit in both radiological progressions and overall survival is statistically significant [15]. Conversely, this assumptions falls down for sunitinib in advanced renal cell carcinoma, where patients receiving such drug show a dramatic difference in responses when compared to interferon, with no difference in disease stabilization [16]. Besides, the benefit is confirmed with much more efficiency in progression-free-surivival and in overall-survival in the censored analysis, taking into account the cross-over [16,17]. The mentioned assumption is again to be considered as false if patients are selected on the basic of molecular features. A phase II study conducted to test the activity of erlotinib in advanced pretreated NSCLC patients displaying the mutation of the EGFR gene, shows an overall response rate of 82%, ten-fold greater of what we are used to see in such setting if not selected on the basis of molecular features [18]. Although this is a phase II study, these data are impressive.

## Phase II randomized studies: a new tale with targeted agents

One other bias of single-arm classical phase II is that the obtained response rate could be better owing to the patient selection (even when the historical benchmark border is correctly chosen). How this problem could be overcome? A solution is offered by randomized phase II, where, according to selection design, multiple experimental drugs or regimens are concurrently tested together, and the winner (with regard to the outcome) is 'picked' and proposed for the further phase III study. These studies are currently misinterpreted due to not-allowed or comparison, although not adequately dimensioned for any survival (or other efficacy outcome) difference. The overall number of such studies has significantly increased with the introduction of new drugs, as reported in the analysis performed by El-Maraghi et al, in which is reported that overall response are still used as activity parameter for molecular agents, and it is predictor of success in phase III, in a series of 89 studies [19]; 30% of such studies are designed in a randomized fashion.

So far, the randomized phase II trial had to: 1) test experimental drugs or combination, and pick the winner for further phase III; 2) be aimed to safety and activity (i.e. response rates); 3) do not use survival end-points; and finally 4) never compare treatment arms. What about new molecularly targeted agents from now on? The issue should be approached balancing risks and benefits between two options. If we use the randomization as a control tool, the question is: in order to obtain more accurate results from early studies with molecularly targeted agents, what is less dangerous? An uncontrolled single-arm phase II, with response as end-point, or a controlled multiple-arm randomized phase II, with survival (or similar efficacy parameter) as end-point. Taking into account the issues raised by Ratain et al [11], uncontrolled designs (i.e. 'classical' phase II), have high efficiency in identifying non-active drugs (high negative predictive value), but low efficiency in selecting the best challengers for phase III (low positive predictive value), while controlled designs (i.e. 'comparative' phase II randomized) have increases positive predictive value, should be (must be) conducted with permissive statistical error criteria (higher alfa-error), and must be followed (if positive) by a classical phase III with traditional rules.

Recently, some authors have encouraged randomized design for phase II trials, to allow a formal comparison between experimental and standard treatment. This should lead to a better interpretation of the results obtained with the experimental treatment that are in most cases difficult to interpret in the absence of control. Of course, the adoption of a randomized design should not transform a phase II into a phase III trial, because the latter is characterized by more stringent criteria, requiring a sample size that would be too large and inappropriate for the early evaluation of an experimental treatment. Randomized phase II trials could instead be conducted according to so-called 'relaxed' criteria, with a power not exceeding 80% and one-tailed alpha error set to 15% or 20%, much higher than commonly accepted [11,20]. Such a high risk of false positive results, which would be of course unacceptable in a phase III trial, can be acceptable in this early context, leading to small sample sizes, to quickly select promising treatments that will be subsequently tested for efficacy. According to some authors, randomized phase II trials may become the standard approach for development of targeted drugs and should be designed with explicit comparative intent [21]. A peculiar type of randomized phase II trial is the so-called "randomized discontinuation design" (RDD) [22,23]. After a first stage in which all patients receive the experimental drug, in the second stage only patients with stable disease are randomized to receive placebo or the active drug. RDD was created with the aim of better interpreting the cause/effect relationship between drug administration and disease stabilization, which is potentially related to treatment-induced growth delay and to enrich the study population for responsive subjects. In the RDD, the comparison between patients shifting to placebo and patients continuing the drug should allow to understand whether the stabilization achieved in those patients was simply related to the natural history of disease or due to treatment activity.

## Targeted agents: moving to phase III trials

Moving to phase III trials with new molecularly targeted agents, few considerations must be done: the vast majority of cancer therapies do benefit only a patient' subgroup between all patients those are administered. If we will be able to target treatment upon the right patients we will maximize the benefit of treated patients, we will provide treatments more cost-effective for the entire society, and finally (but more relevant for clinical research) we will get more informations for successful clinical trials.

The vast majority of informations regarding the eventual preferential effect of a molecularly targeted agents on a specific molecular features, whatever it is, mutation, overexspression or amplification, is provided by retrospective analyses of large randomized trials exploring the benefit of the adopted new drugs into a unselected population. Thereafter, subgroup analyses (mainly unplanned) are performed, and, for those characteristics requiring tissue and/or blocks, these are done on even small samples, i.e. in those patients where the tissue is available. With these perspectives, it sees rather obvious that any conclusions should be softened are weighted with the real statistical power of the original analysis which the trial is design for.

The results of the recent trial exploring the effect of cetuximab over best supportive care (BSC) in advanced pre-treated colorectal cancer patients according to the k-RAS gene mutation are consistent with those recently presented at the last ASCO meeting, which restrict the benefit of cetuximab to wild-type patients [24-26]. k-RAS status seem to not have any prognostic role in OS in patients receiving BSC, while in the trial recently published by Amado et al, a prognostic effect of the k-RAS status is present in the BSC arm in comparison to panitumumab [27]. These data stress the controversy in the data interpretation process of retrospective analyses for clinical practice.

Nevertheless, conducting a phase III trial in the traditional manner without strict eligibility criteria may result in a false negative trial, unless a sufficiently large part of the treated patients have tumors were by the target is expressed. So, the more the target is underrepresented in the original sample, the more the chance to find right answer decreases. Greater emphasis should be probably given, when planning a clinical trial and when interpreting its results, to the great impact that the molecular heterogeneity of tumors, affecting sensitivity to the experimental treatment, may have on the results of a clinical trial [28]. This concept has been never taken into account in the planning and the analysis of clinical trials with cytotoxic agents, but it should be necessarily considered in clinical trials with molecularly targeted agents. In a simplified situation, in which the whole population of patients is divided in two distinct genotypes (A and B) – where genotype A is characterized by sensitivity to the experimental treatment producing in this group an outcome better than in the control group, and the genotype B is characterized by absence of difference in efficacy between experimental and standard treatment – the higher the proportion of patients with genotype B in the study sample, the lower the power of the clinical trial to show a positive result. The statistical power of the study is even lower if we postulate that the genotype B determines a detrimental effect of experimental treatment compared to control. Also in the case that the targeted population is well represented, and the trial gives positive results in favor of the new drug, this means that this effect is driven by that subset of patients, anyway administering the treatment to many patients who do not really benefit.

Moreover, the subgroup analysis process itself is biased by many risks of data distortion. According to the brilliant paper published by Lagakos et al, if you test 10 subgroups, your chance to occur into more than 3, more than 2, and more than 1 false positive results is around 2%, 9% and 40% [29].

Any 'Post hoc' exploratory subgroup analyses (i.e. the comparison of experimental and standard treatment separately in subgroups of patients identified by the biomarkers status, without a priori planned hypotheses) is a dangerous procedure, because of the high risk of both false positive and false negative results [30]. Importantly, comparison of treatment and control should not be performed separately in each subgroup, but formal test of interaction should be performed [30]. Of course, results of tests for interactions are likely to be convincing only if they were specified at the start of the study. In any study that presents subgroup analyses it is important to specify when and why the subgroups were chosen [30,31]. With all these considerations, the risk of mis-interpretation of subgroup analyses, which is high by itself, does increase when molecular characteristics are approached.

With regard to the last point, prospectively specified analysis plans for randomized phase III studies are fundamental to achieve reliable results. Paradoxically, many of the currently ongoing trials for adjuvant treatment of resected NSCLC are designed in order to select patients on the basis of genetic features when 'old-fashioned' chemotherapeutics are experimented (i.e. the Spanish Customized Adjuvant Treatment, SCAT, randomizing patients on the basis of BRCA overexpression, the and the International TAilored Chemotherapy Adjuvant trial, ITACA, with a two-step randomization taking into account both levels of ERCC1 and TS tissue expression), and with a non-selection strategy, when adopting 'new and targeted' agents (i.e. erlotinib and bevacizumab in the RADIANT, and in the ECOG E1505 trial, respectively).

In an ideal scenario, when complete information on predictive factors and proper selection of patients can be definitely obtained in the early phases of drug development, the conduction of subsequent phase III study could be optimized. Unfortunately, this ideal scenario occurs rarely, also with molecularly targeted agents. When planning a phase III trial comparing an experimental treatment with the standard, we often have evidence supporting a predictive role of a marker (M) about the efficacy of the experimental treatment: according to that evidence, patients with expression of the marker (M+) are expected to potentially benefit of the experimental treatment, and patients with absence of expression of the marker (M-) are not [32]. In such a scenario, different strategies based on prospective determination of marker status are theoretically possible: (a) "*randomize-all*" strategy, randomization between standard and experimental treatment without selection, bu with stratification based on the status of the marker; (b) "*targeted*" design, randomization between standard and experimental treatment only in patients selected according to the status of the marker; (c) "*customized*" strategy (also called "*marker-based strategy*"), randomization between standard arm, in which the treatment is the same for all patients, and a personalized arm, in which treatment is chosen based on the marker status of each patient.

The "*randomize-all*" strategy is useful if investigators are not sure of the complete lack of efficacy of experimental treatment in M- patients. Marker is prospectively assessed in all patients, allowing stratification, but all patients are randomized, regardless of the marker status. Interaction between marker status and treatment effect can be formally tested by an interaction test. On the contrary, predictive role of the marker should not be addressed with separate comparison in M+ and M- patients, because this approach, as stated before, would be associated with a high risk of false results [29].

An alternative strategy ("*targeted design*") is to test the status of the marker M, randomizing only M+ patients. This strategy is acceptable only in cases where investigators have already enough evidence to completely rule out the efficacy of the experimental treatment in M- patients. Due to the absence of M- patients, targeted design allows investigators to avoid potential dilution of the results.

A third approach is the so-called "*strategy design*". According to this design, the experimental arm will receive a personalized treatment based on the status of predictive marker, while all patients assigned to the control arm receive standard treatment. A great limit of strategy design is related to the proportion of M+ patients on the overall number of patients. If M+ patients are a small minority, treatment received will be nearly the same in both arms, and the study will provide little information on the efficacy of experimental treatment. On the contrary, the strategy design will be particularly effective when both M+ and M- patients represent a significant proportion of the patients.

## Conclusion

The success of a targeted drug development (and the patient benefit) strongly depends on extensive pre-clinical and early clinical modeling, and so depends on conducting good science. Early phases, and in particular phase II studies, remain crucial for development of targeted drug, because this is the moment in which it is possible to explore surrogate and potential selection biomarkers.

With these intents, phase II trials should be hypothesis-generating and should signal either to progress to phase III, and to go back to the lab. How clinical trial design with molecularly targeted agents should be improved and fasten to realize the real 'bench to bedside' medicine? Molecularly targeted agents should be studied with those early phases with the newest adaptive design [17], with a more realistic basic hypotheses [33], and be 'tailored' on a clearly specific molecular feature or signaling [34]. This pivotal process, will come up into more accurate early studies, providing few positive studies but with stronger and more reliable results. Few drugs will enter the phase III fashion, by increasing the chance to win over the standard. These following phase III trials (which remain always mandatory), will be able to test more frequently superiority hypotheses, providing big differences, less patients to be enrolled, into shorter time for completing the studies.

## Competing interests

The authors declare that they have no competing interests.

## Authors' contributions

EB, MDM and MM planned and conceived the review; EB, MDM, FC and MM carried out all available evidences; EB, MDM, FC, DG, FC, PC, and MM drafted the manuscript; all authors read and approved the final manuscript.
